# A Novel Method for Heat Haze-Induced Error Mitigation in Vision-Based Bridge Displacement Measurement

**DOI:** 10.3390/s24165151

**Published:** 2024-08-09

**Authors:** Xintong Kong, Baoquan Wang, Dongming Feng, Chenchen Yuan, Ruoyu Gu, Weihang Ren, Kaijing Wei

**Affiliations:** School of Civil Engineering, Southeast University, Nanjing 211189, China; xintongkong@seu.edu.cn (X.K.); dfeng@seu.edu.cn (D.F.); 213210335@seu.edu.cn (C.Y.); 213210097@seu.edu.cn (R.G.); 213212125@seu.edu.cn (W.R.); 213213758@seu.edu.cn (K.W.)

**Keywords:** computer vision, displacement measurement, heat haze mitigation

## Abstract

Vision-based techniques have become widely applied in structural displacement monitoring. However, heat haze poses a great threat to the precision of vision systems by creating distortions in the images. This paper proposes a vision-based bridge displacement measurement technique with heat haze mitigation capability. The properties of heat haze-induced errors are illustrated. A dual-tree complex wavelet transform (DT-CWT) is used to mitigate the heat haze in images, and the speeded-up robust features (SURF) algorithm is employed to extract the displacement. The proposed method is validated through indoor experiments on a bridge model. The designed vision system achieves high measurement accuracy in a heat haze-free condition. The proposed mitigation method successfully corrects 61.05% of heat haze-induced errors in static experiments and 95.31% in dynamic experiments.

## 1. Introduction

The structural displacement under dynamic loads is a crucial indicator for evaluating the health status of bridges, as it directly reflects the overall structural stiffness. Displacement measuring systems currently employ contact (e.g., accelerometer, linear variable differential transformer (LVDT)) [1,2] and non-contact (e.g., GPS, laser vibrometer) [3,4,5] sensors to obtain the structure’s dynamic displacements. However, these sensors suffer from many limitations in field application. For instance, contact sensors require physical access to the structure for installation, and a stationary point is needed for reference, which is often difficult or even impossible to locate [6]. GPS sensors are easier to install, but the measurement accuracy is limited, usually with errors ranging from 5 mm to 10 mm [6]. The laser vibrometer is generally accurate, but it is not only costly but also limited in measuring distance (typically less than five meters) [7].

The emergence of vision-based displacement measuring techniques has brought new prospects to the non-contact, high-precision, long-distance, and multi-target monitoring of civil structures. Unlike conventional sensors, vision-based sensors do not need a stationary platform. With long-focus lenses, vision-based sensors can be deployed hundreds of meters away from the target structure. Vision-based measuring systems have been successfully used in monitoring the displacements of bridges [5,6], frame structures [8,9], and other civil and industrial structures [3,10].

In practice, the accuracy of vision-based sensors is easily affected by environmental factors (e.g., light, rain, fog), among which heat haze poses great challenges to the precision of monitoring in hot weather [11]. Heat haze occurs when the air is heated unevenly by a high ambient temperature. The nonuniformly heated air causes variations in its optical refraction index, resulting in image distortion. The distortion caused by heat haze will be mixed with real displacements collected by the vision-based sensor, which induces measurement errors. Additionally, the measurement errors caused by heat haze increase as the distance increases due to more air in between, which can reach as large as 50 mm [12]. Hence, it is vital to mitigate heat haze-induced errors to ensure high accuracy and long-term monitoring.

Research on the elimination of heat haze-induced errors generally falls into three categories. The first category employs hardware (e.g., pneumatic device [13], optical filter [14]) either to homogenize the air or to increase exposure time so that heat haze-induced errors can be neutralized. Such techniques are restricted to small-scale monitoring due to limitations in the size and cost of devices. The second category uses lucky imaging [15], image fusion [16], space-invariant deconvolution [17], etc. to reconstruct images free of heat haze. These methods recover one single image from a series of images, which makes real-time monitoring impossible. Moreover, with heat haze-induced errors eliminated, real structural displacements will also be eroded. In the third category, the statistics of heat haze are studied and used to re-warp distorted frames back to a heat haze-free version [18]. However, it requires a base of heat haze-free images for every target, making this method inappropriate for wide applications. In addition, Anantrasirichai et al. [19] used a Gaussian mixture model (GMM) and Kalman filtering to track moving objects in heat haze environments. However, this method’s high computational complexity necessitates significant processing resources, posing challenges for real-time monitoring. Deledalle et al. [20] incorporated a physical model, the Fried kernel, to represent the impact of atmospheric turbulence on optical resolution. Nevertheless, the static kernel may struggle to differentiate real movements from blurs caused by heat haze, limiting its recovery accuracy. The alleviation technique that Luo et al. [21] designed can successfully correct heat haze-induced errors, but it requires a static background object for reference. Thus, this technique can only apply to certain scenarios. In conclusion, there is currently a lack of a straightforward, universal, and real-time mitigation method for heat haze-induced errors in the dynamic displacement monitoring of bridges.

In light of the aforementioned literature, this study proposes a novel vision-based method for extracting real-time displacements of a bridge structure in a heat haze environment. Firstly, the camera is calibrated to obtain physical displacements from pixel displacements. Secondly, the dual-tree complex wavelet transform (DT-CWT) [22] is used to pre-process the images captured by the camera. Thirdly, a feature detection algorithm, speeded-up robust features (SURF) [23], is employed to obtain displacements between frames. Finally, the raw displacements undergo a two-step refining process to improve measurement accuracy. The novelty of the proposed method lies in the instant recovery of distorted images, the high elimination rate of heat haze-induced errors, and the possibility of real-time monitoring.

The paper is organized as follows: In Section 2, the framework of the proposed method is elaborated. Properties of heat haze-induced errors are investigated, and procedures for heat haze detection, image recovery, and displacement acquisition are presented. In Section 3, the proposed method is validated through lab experiments with man-made heat haze, and results under different parameters are discussed. Finally, the conclusion is drawn in Section 4, along with the future research prospects.

## 2. Framework of the Proposed Method

**Figure 1:**
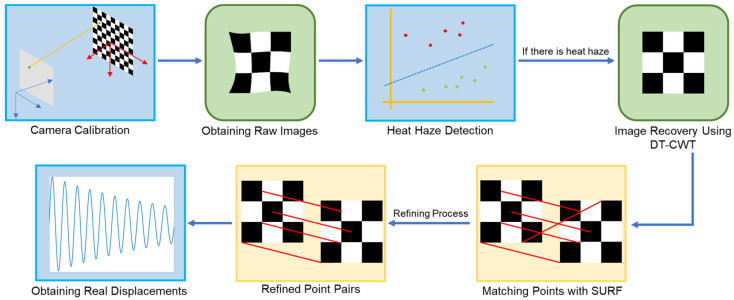
Overall flowchart of the proposed method.

In this section, the framework of the proposed method is presented. Firstly, the principle of camera calibration is elaborated. Secondly, the properties of heat haze-induced errors are introduced. Thirdly, methods of heat haze detection and feature detection are explained. Finally, the heat haze mitigation technique is detailed, along with the displacement extraction and refinement processes. Figure 1 shows the overall flowchart of the proposed method.

### 2.1. Camera Calibration

Camera calibration aims to transform pixel displacements into physical displacements. Parameters including camera intrinsics, camera extrinsics, and distortion coefficients [24] are estimated so that the world coordinates of any point can be reconstructed from its image coordinates. When the optical axis of the camera is perpendicular to the object’s surface, all points on this surface have a uniform depth of field, meaning that these points can be equally scaled down into the image plane. In this case, only the scaling factor (SF) is needed. If the physical distance and corresponding pixel distance of two given points on the target are known, the SF can be calculated using Equation (1) [6].

(1) $$\mathrm{SF}=\frac{d_{\mathrm{known}}}{I_{\mathrm{known}}}$$

where $d_{\mathrm{known}}$ is the physical distance and $I_{\mathrm{known}}$ is the pixel distance, which can be obtained through computer processing.

It should be noted that, when the optical axis of the camera is not perpendicular to the object’s surface, more complicated calibration processes will be needed to reconstruct world coordinates from image coordinates. For instance, the angle at which the optical axis is tilted in the normal direction of the object’s surface needs to be known. Detailed calibration procedures are also described in Ref. [6].

### 2.2. Properties of Heat Haze-Induced Errors

Heat haze occurs when density variations in the air, caused by heat, alter its optical refraction index, resulting in distortions in video images. Studies have demonstrated that heat haze can induce blurring, dithering, offset, and random noise in images [25,26]. Unlike fog, which blurs the image mainly by altering pixel intensity [27], heat haze generates a “shearing effect”, where different parts of the object move in different directions [16]. Moreover, the direction and magnitude of the distortion at a specific point vary over time. The spatial and temporal-variant distortion effect makes a model-based solution difficult to achieve [19].

### 2.3. Heat Haze Detection Technique

A heat haze detection technique is proposed to judge whether an image is subject to heat haze. It has been demonstrated that heat haze interferes with an image primarily by increasing the spatial variance of the image [18]. Given this, a detection technique based on support vector machines (SVMs) is developed, derived from the approach described in Ref. [28].

The fundamental principle of an SVM is to find the optimal hyperplane that best separates the data points of different classes in a high-dimensional space. For a binary classification problem, the hyperplane can be described by the equation $\omega^{T}x+b=0$, where **ω** is the weight vector, **x** represents the input features, and b is the bias term. The vector **ω** and the scalar b are the parameters of the model, which are to be calibrated through the training dataset.

The objective of the SVM classifier is to optimize the separation between the support vectors and the hyperplane. The proximity between the support vectors and the hyperplane is determined by the expression:

(2) $$d=\frac{\left|\omega^{T}x+b\right|}{\left\|\omega \right\|}$$

Consequently, the goal is to maximize the value of d. Since ${|\omega }^{T}x_{i}+b|=1$ for support vectors $x_{i}$, the target distance d can be expressed in a simplified form as follows:

(3) $$d=\frac{1}{\left\|\omega \right\|}$$

Therefore, the objective function of the classifier can be drawn as follows:

(4) $$f(\omega )\;=\frac{1}{2}||\omega {\left|\right|}^{2}$$

The aim of optimization is to find the arguments that minimize this function.

In this context, the features used to train the classifier are the spatial variances of intensity along the x, y axes in an image. An image is judged to be without heat haze if $\omega^{T}x+b\leq 0$. If $\omega^{T}x+b\;>\;0$, the image is judged to be subject to heat haze.

### 2.4. Feature Detection: Speeded-Up Robust Features

The proposed method uses feature detection to extract displacements between frames. Feature detection identifies points of interest in an image with distinctive features, such as corners (sharp features) or blobs (smooth features) [11]. SURF is recognized as a fast, accurate, and robust feature detection algorithm [23]. It is designed as an improvement over scale-invariant feature transform (SIFT) [29], aimed at increasing processing speeds while maintaining high quality and reliability in feature detection.

SURF employs a Hessian matrix-based method to detect interest points, strategically departing from SIFT’s Laplacian of Gaussian approach. This innovation not only accelerates the detection process but also strengthens the algorithm’s capacity to handle images with blurring and rotational variations [30]. For a point **x** = (x, y) in an image I, the Hessian matrix H(**x**, σ) in **x** at scale σ can be expressed as:

(5) $$H\left(x,\sigma \right)=\left[\begin{array}{cc} L_{\mathrm{xx}}\left(x,\sigma \right) & L_{\mathrm{xy}}\left(x,\sigma \right)\\ L_{\mathrm{xy}}\left(x,\sigma \right) & L_{\mathrm{yy}}\left(x,\sigma \right) \end{array}\right]$$

where $L_{\mathrm{xx}}\left(x,\sigma \right)$ is the convolution of the Gaussian second-order derivative $\frac{\partial^{2}}{\partial x^{2}}g(\sigma )$ with the image I at point **x**, and similarly for $L_{\mathrm{xy}}\left(x,\sigma \right)$ and $L_{\mathrm{yy}}\left(x,\sigma \right)$. The determinant of this matrix can be calculated as:

(6) $$\det\left(H\right)=L_{\mathrm{xx}}L_{\mathrm{yy}}-{\left(L_{\mathrm{xy}}\right)}^{2}$$

This determinant helps pinpoint areas where the response to the Hessian determinant is maximal, suggesting potential feature-rich regions. The descriptor for each interest point is then constructed by summing Harr wavelet responses using integral images [31]. The dominant orientation is determined from the sum of all wavelet responses within a sliding orientation window, calculated as:

(7) $$\theta =\Sigma \arctan\left(\frac{\Sigma |\mathrm{dy}|}{\Sigma |\mathrm{dx}|}\right)$$

After establishing the orientation, the descriptor is crafted by aligning a square region to this orientation and dividing it into smaller 4 × 4 sub-regions. Within each sub-region, the descriptor accumulates sums and absolute sums of Haar wavelet responses in both horizontal and vertical directions:

(8) v=(Σdx,Σdy,Σ|dx|,Σ|dy|)

SURF is scale-invariant and rotation-invariant and proves robust in image deformation and lighting variations [32], making it suitable for analyzing images captured from multiple viewpoints or varying significantly in appearance. SURF also excels in dynamic and time-sensitive scenarios, so it is a proper method for monitoring displacements. The detailed displacement calculation procedures will be elaborated on in Section 2.6.

### 2.5. Heat Haze Mitigation

In the proposed method, DT-CWT is performed to mitigate the heat haze in an image. DT-CWT has been widely applied in image fusion, where useful information from several source images is selected and combined into a new image [33,34,35].

Several properties enable DT-CWT to restore heat haze-distorted images. Firstly, DT-CWT is near-shift invariant so that it can identify and locate local distortion in an image accurately, regardless of its specific location. Secondly, DT-CWT has good directional selectivity, allowing it to recognize and process distortion from different directions. Thirdly, the Hilbert transform used in DT-CWT facilitates the analysis of local frequency information and thus enables it to distinguish real displacements from interference. Lastly, the phase of a DT-CWT coefficient is robust to noise and temporal intensity variations, making it effective for removing distorting ripples [16].

**Figure 2:**
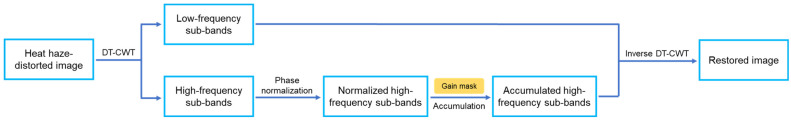
The heat haze mitigation process.

Previous studies [16,19] have used DT-CWT to mitigate heat haze in images. A sequence of heat haze-distorted images is analyzed through a region-based process to restore one single image. Hence, these methods cannot achieve real-time processing. This study proposes a pixel-based method that only requires the target image itself for it to be restored. The region-based method segments the image into regions and processes each region separately, and the fusion strategy is more complex. In contrast, the pixel-based method directly combines pixel values with a simpler averaging operation. Therefore, the pixel-based method can achieve faster processing. The detailed mitigation process is as follows: for a heat haze-distorted image, DT-CWT is performed iteratively on it to obtain high-frequency and low-frequency sub-bands. In this paper, the number of iterations of DT-CWT is denoted as level, which is a significant influence parameter, and the impact of its different values on the experimental results will be discussed in Section 3.6. During the iteration process, the low-frequency sub-bands are preserved intact, while the high-frequency sub-bands undergo a phase normalization process. Then the weighted sum of high-frequency sub-bands is calculated through gain masks. Finally, the inverse DT-CWT is performed to reconstruct the processed image. Figure 2 shows the flowchart of the mitigation process.

### 2.6. Displacement Extraction and Refinement

The SURF algorithm is utilized to extract displacements between frames. To calculate the displacement between the ith frame and the reference frame (the 1st frame in this context), the first step is to detect SURF points in the reference frame. Afterwards, the descriptor, also known as the feature vector [23], for each SURF point is extracted. The ith frame then undergoes the same process. These two sets of SURF points are matched by searching for feature vectors with a minimum Euclidian distance. The displacement between the two frames can be determined using the displacements of matched point pairs. The above-mentioned procedures can be accomplished with open-source MATLAB R2022 functions.

The direct idea is to use the mean displacement of all point pairs as the integrated displacement between the ith frame and the reference frame. However, since the searching mechanism of SURF is largely based on local intensity [11], mismatches may occur where patterns are alike in shape and intensity. Worse still, mismatched points usually have exceptionally large or small displacements. Considering that the mean is significantly affected by extremes, the elimination of mismatched points is especially necessary.

**Figure 3:**
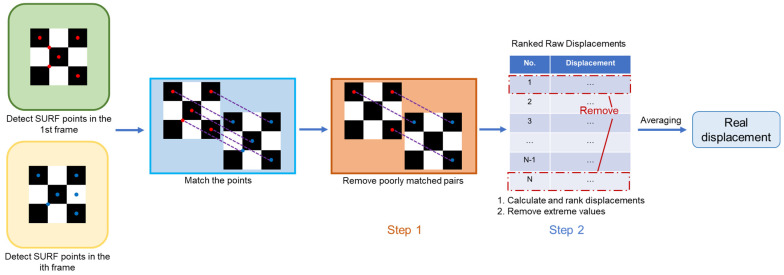
Displacement extraction and refinement processes.

In the proposed method, a two-step refining process is developed to eliminate mismatched points. In Step 1, the matched point pairs are sorted according to the sum of the absolute difference (SAD) of the feature vectors. A subset with the smallest SADs (best-matched points) is chosen to calculate the overall displacement. After that, the displacements between each selected point pair are determined and indicated as the raw displacements. In Step 2, an extremum removal mechanism is implemented. The raw displacements are ranked, and portions with the highest and lowest values are removed. Finally, the real displacement is obtained by calculating the mean of the remaining displacements. The percentage of removal is referred to as the removal threshold in the rest of the paper. If the removal threshold is set to 10%, it means that the largest 10% and smallest 10% of all raw displacements will be removed. Figure 3 shows the processes of displacement extraction and refinement.

## 3. Experimental Validation and Discussion

Lab experiments are conducted to validate the proposed method. Static and dynamic tests are performed, and displacement data obtained by the vision system are compared to those collected by a laser displacement sensor (LDS). Heat haze-induced errors are first demonstrated and then mitigated by the proposed method. Different parameters are finally discussed in terms of their influences on the mitigation effect.

### 3.1. Experimental Setup

**Figure 4:**
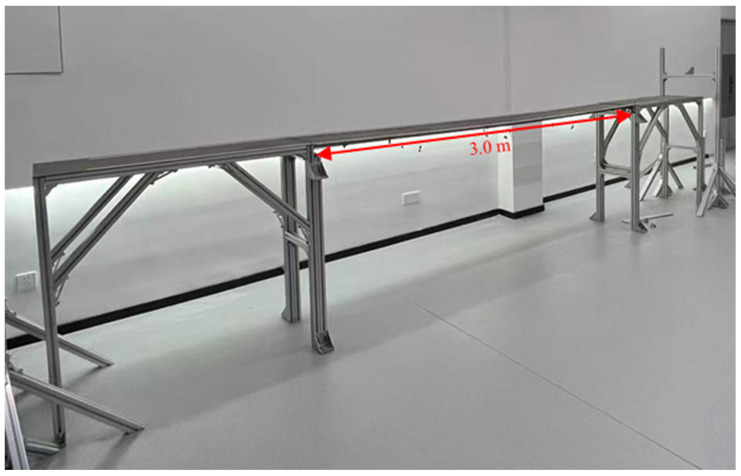
Bridge model.

A scaled bridge model is used as the monitoring target, as shown in Figure 4. The main span of the model is a simply supported system with a roller and a pin at both ends. The deck of the main span has a length of 3.0 m and a width of 0.4 m, and it is 1.08 m above the ground. Details of the model are described in a previous study [4]. A square paper marker measuring 4 cm × 4 cm is stuck to the side of the deck, and it is placed at the longitudinal midpoint. The marker is adjusted to keep its plane vertical. The camera is calibrated based on the length of the target. The scaling factor herein is 0.2006 mm/pixel.

The vision system consists of a mini SLR camera with a lens, a tripod, and a computer. The camera is a Nikon Z30 (Nikon Corporation, Tokyo, Japan) with a CMOS sensor and a resolution of 1920 × 1080. The lens is an AF-S Nikkor 18–140 mm (Nikon Corporation, Tokyo, Japan). The computer is equipped with an Intel(R) Core i5-1135G7 CPU at 2.40 GHz and 16.0 GB of RAM (Intel, Santa Clara, CA, USA). In the experiments, the camera is fixed on the tripod at the same height as the marker, and the optical axis is set to be horizontal. This step is to ensure that the optical axis is perpendicular to the marker’s plane. The LDS used in the experiments is BOJKE BLG-85N-485 (BOJKE, Dongguan, China) with a measuring precision of 10 μm. More detailed parameters are shown in Table 1.

**Figure 5:**
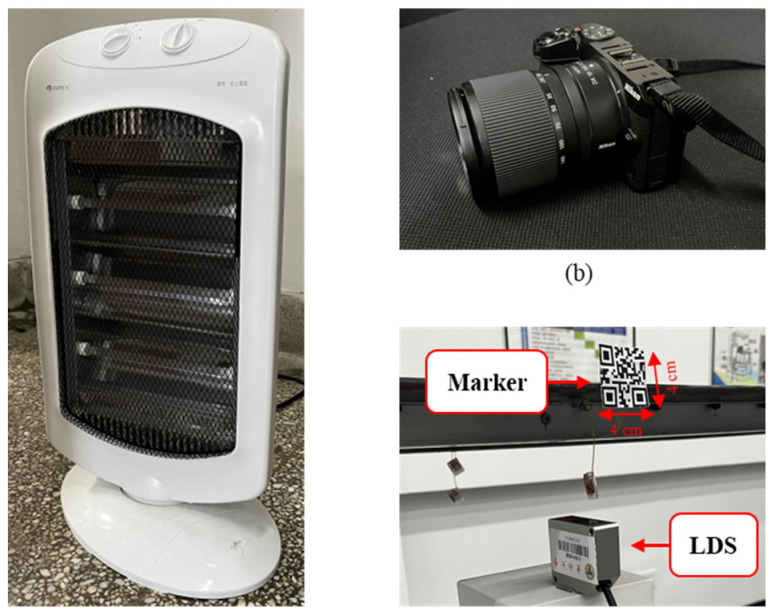
Experiment equipment: (**a**) furnace, (**b**) camera, (**c**) marker, and LDS.

**Figure 6:**
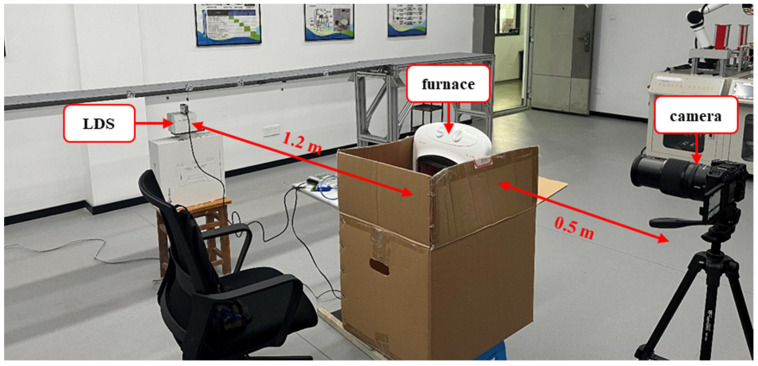
Setup of the experiments.

In the experiments, the heat haze is created by a household electric furnace, as shown in Figure 5a. The furnace is placed between the model bridge and the camera in the light path so that images captured by the camera are distorted by heat haze. The LDS is fixed on a stationary platform right under the marker to ensure that it accurately collects the marker’s displacements. The setup of the experiments is shown in Figure 6.

The experiments were conducted in Nanjing, China, at an altitude of approximately 28 m. The time was December 2023, and the indoor temperature was 15 °C when the furnace was off. After the furnace was heated at 1200 W for five minutes, the temperature at the light path above it was 60 °C.

### 3.2. Validation of the Displacement Measurement Technique in a Heat Haze-Free Environment

**Figure 7:**
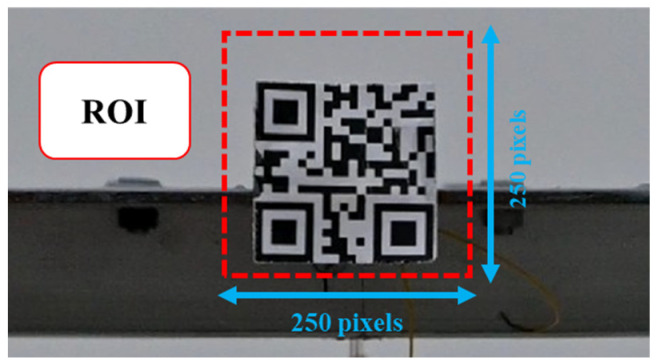
ROI in the test (enclosed in the red square).

In this section, the designed vision-based displacement monitoring technique is validated through a dynamic test without heat haze. The bridge model is excited by an initial displacement in the Y direction and then is allowed to vibrate freely. The camera records the video of the vibrating target while the LDS collects the displacement data. The video is then transmitted to the computer for processing. The displacements obtained by the vision system are compared to those extracted by the LDS. The region of interest (ROI) is set to be a 250 × 250 pixel area enclosing the square pattern, as shown in Figure 7.

**Figure 8:**
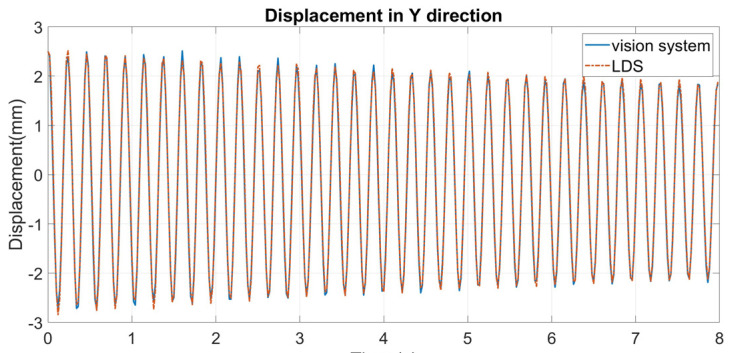
Dynamic displacements extracted by the vision system and the LDS without heat haze.

An eight-second sample is used for comparison. The results are shown in Figure 8. It can be seen that, despite a few discrepancies near the peaks of the curve, the displacements extracted by the vision system match well with those extracted by the LDS.

The root mean square error (RMSE) is employed to assess the accuracy of the vision system.

(9) $$R\mathrm{MSE}\;=\sqrt{\frac{1}{n}\sum_{i=1}^{n}{\left(Y_{i}-{\hat{Y}}_{i}\right)}^{2}}$$

where n is the number of time points, ${\hat{Y}}_{i}$ is the displacement measured by the vision system, and $Y_{i}$ is the corresponding displacement measured by the LDS (considered the real value here). The RMSE of the displacements obtained by the vision system is 0.0847 mm. This demonstrates that the designed vision system can accurately measure the dynamic displacements of a bridge.

In the experiments, only one type of marker (one with a Quick Response code pattern) is used as the target. Since SURF detects points that are distinct in local intensity, such as corners and blobs, a suitable marker should contain a clear pattern with high contrast. Moreover, the marker should not be a simple array of similar patterns (like a mosaic), as mismatches will happen in this case. Additionally, the marker should be large enough to provide sufficient SURF points.

### 3.3. Illustration of Heat Haze-Induced Errors

In this section, heat haze-induced errors are illustrated through static and dynamic tests. In the static test, the furnace is first heated at 1200 W for five minutes. Then, the camera starts to record a video of the target, which remains immobile. It should be noted that since the target is itself static, the true displacements of the target equal zero. Any displacement detected by the vision system is considered an error caused by heat haze.

**Figure 9:**
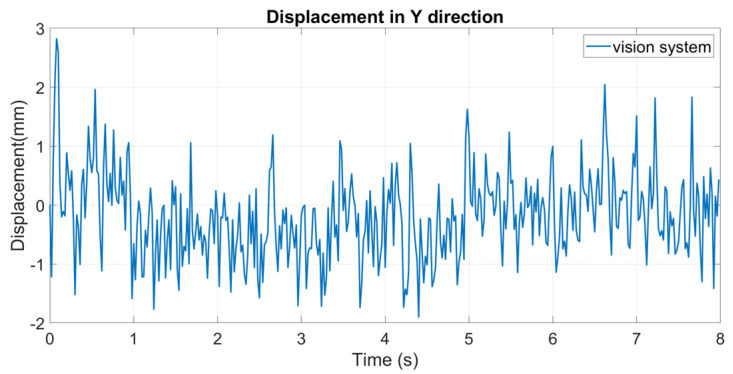
Displacement errors in the static test.

An eight-second sample is selected for analysis. The result is shown in Figure 9 (RMSE = 0.7564 mm). As can be seen clearly, the displacements extracted by the vision system are seriously distorted by heat haze. Random fluctuations occur in the displacement curve, which proves that the distortion caused by heat haze is a temporal variant.

**Figure 10:**
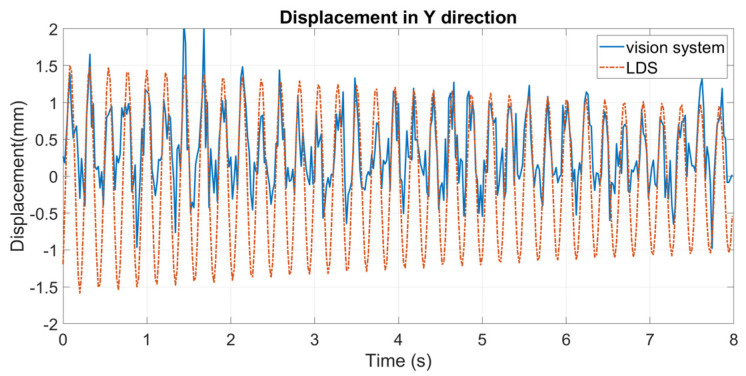
Dynamic displacements extracted by the vision system and the LDS with heat haze.

In the dynamic test, the bridge model is excited by an initial displacement in the Y direction and then is allowed to vibrate freely. The other settings are the same as the static test. The result is shown in Figure 10 (RMSE = 0.7248 mm). The two tests demonstrate that heat haze can bring tremendous error to the vision system in both static and dynamic conditions.

The spatial-variant and temporal-variant properties of heat haze are illustrated through another static test. In a continuous image sequence (time interval = 0.1 s) subject to heat haze, motions between successive images are detected. Herein, three consecutive images (images 1, 2, and 3) are chosen. After that, movements between images 1 and 2 and 2 and 3 are determined, respectively.

**Figure 11:**
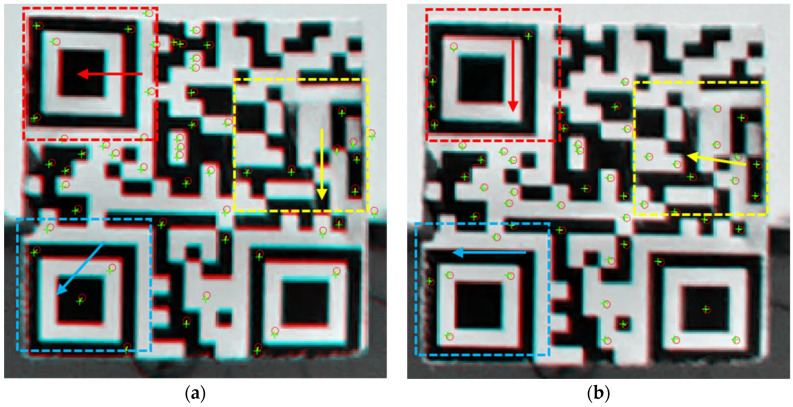
Movements between images 1 and 2 (**a**) and between images 2 and 3 (**b**).

The results are shown in Figure 11, where red circles represent SURF points in the former image and green plus signs represent those in the latter image. In the top left corner, enclosed by a red square, image 2 has a leftward movement relative to image 1, whereas image 3 has a downward movement relative to image 2. Simply looking at the movement between images 1 and 2, it can be seen that different regions move in different directions. Thus, this test demonstrates that heat haze-induced errors are both spatial-variant and temporal-variant.

### 3.4. Heat Haze Detection

This section introduces the training and testing of the heat haze detection model described in Section 2.3. The dataset used for training and testing the model is images of the marker shown in Section 3.1. Images with heat haze and those without are classified and labeled manually. The training set consists of 1324 negative samples (without heat haze) and 1540 positive samples (with heat haze), totaling 2864 samples. The model is assessed by the percentage of correctly predicted samples out of the total samples in the testing set, denoted as accuracy. After multiple training sessions, the accuracy of the model reaches 73%. This result shows that this method can successfully judge whether there is heat haze in an image.

It is found in the training process that the model performs better in judging positive samples, while misjudgments happen more frequently in negative samples. Another noteworthy phenomenon is that if the training set contains the pattern or similar patterns of the test image, the possibility of a correct judgment is significantly higher. Therefore, in practical application, it is necessary to add images of the target itself to the training dataset to improve the accuracy of the model.

### 3.5. Validation of the Proposed Heat Haze Mitigation Method

This section validates the proposed heat haze mitigation method. The method is applied to images subjected to heat haze in static and dynamic experiments. The processed images are imported into the vision system once again. The mitigation effect is assessed by comparing the measurement errors before and after mitigation. Here the level of DT-CWT is set to be three and the high-pass gain mask is set to be [2.0, 1.4, 1.0, 1.0]. The best 50 pairs of matched points are used to calculate the mean displacement, and the removal threshold is 10%.

**Figure 12:**
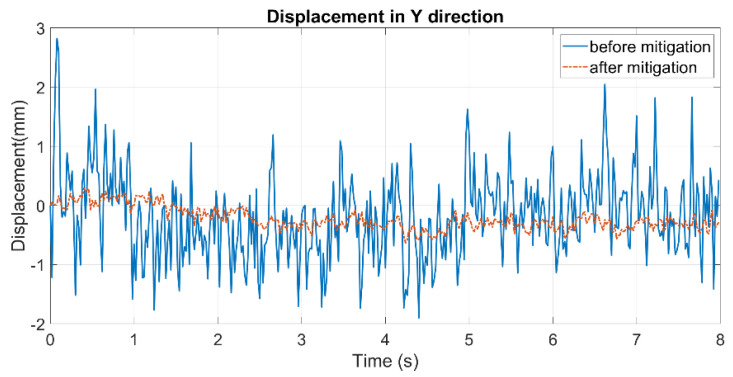
Mitigation effect in the static experiment.

**Figure 13:**
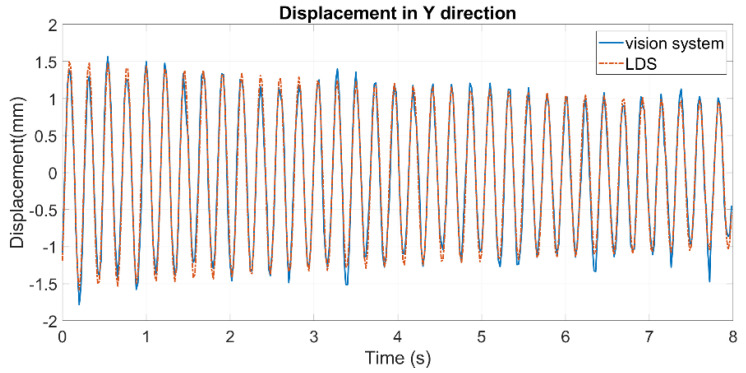
Dynamic displacements extracted by the vision system and the LDS after mitigation.

Figure 12 shows the displacement curves before and after mitigation in the static experiment from the vision system, and Figure 13 compares the displacements from the vision system after mitigation with those collected by the LDS. The RMSEs in the static and dynamic experiments are 0.2946 and 0.1179 mm, respectively. To assess the mitigation effect, a correction rate is calculated to measure the error eliminated by mitigation.

In the dynamic experiment, the error is assumed to consist of two parts: one is induced by heat haze, and the other is inherent in the vision system (e.g., caused by the inadequate resolution of the camera or the short focal length of the lens). Hence, the error caused by heat haze is considered to be the raw error subtracted from the error in the dynamic experiment without heat haze (0.0847 mm).

(10) $$E_{\mathrm{before}}=E_{\mathrm{before}}^{r}-E_{0}\;E_{\mathrm{after}}=E_{\mathrm{after}}^{r}-E_{0}$$

(11) $$correction\;rate=\left(1-\frac{E_{after}}{E_{before}}\right)\times 100\%$$

where $E^{r}$ (either before or after mitigation) is the raw error directly calculated by comparing the displacements, and E (before and after mitigation, respectively) is the refined error caused merely by heat haze. $E_{0}$ is the error in the dynamic experiment without heat haze (0.0847 mm). For the static experiment, the correction rate can be calculated directly from Equation (11), where the displacement is itself considered an error. The results are shown in Table 2. It can be seen from the correction rates that the proposed method can successfully reduce heat haze-induced errors in displacement monitoring.

It should be noted that the correction rate in the dynamic experiment (94.81%) is significantly higher than that in the static experiment (61.05%). A possible explanation is given here. In the dynamic experiment, the wrongly matched pairs are few in number, and it is easier for the two-step refining process to remove the wrong displacements. However, in the static experiment, there are significantly more wrongly matched pairs, and the values of their displacements are more evenly distributed. (This phenomenon may be related to the underlying mechanics of SURF.) Therefore, the refining process can only remove some of the wrong displacements, making the correction rate lower. The authors intend to further investigate this issue in future research.

The experiments described in this section have successfully validated the proposed heat haze mitigation method, as the displacements obtained from the recovered images match the real displacements well. In this study, the effectiveness of the designed vision system is verified on a simply supported bridge model. Nonetheless, it should be emphasized that the system can be widely applicable to other bridge types since the mechanics of monitoring are the same. However, the designed system is still limited in the following three aspects: Firstly, if clear images of the target cannot be obtained, the accuracy of the displacement measurement technique will be undermined. Therefore, the high resolution of the camera must be ensured, and long-focus lenses are necessary for long-distance monitoring. Secondly, due to its underlying mechanics, SURF tends to be less effective in detecting points in images with lower contrast. Thus, the displacement measurement technique is less suitable for a non-target surface (e.g., a plain concrete surface). Thirdly, it is preferable that the heat haze detection model is trained on the target bridge itself to generate more accurate predictions. Attention to these three aspects will contribute to removing the limitations of the proposed method in terms of applicability.

### 3.6. Discussion of Various Parameters

**Figure 14:**
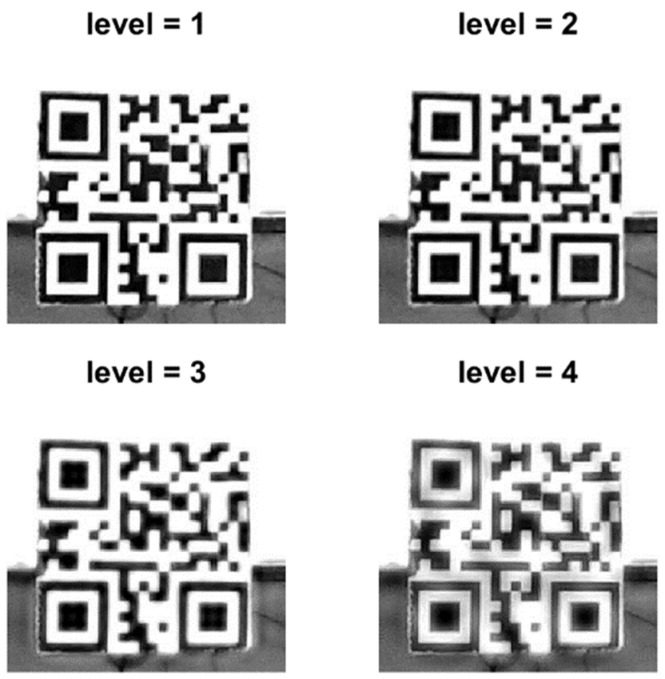
Images of the ROI after heat haze mitigation at different levels of DT-CWT.

After experimental validation of the proposed method, the values of different parameters in the method are adjusted to achieve the best mitigation result. Different levels (levels = 1, 2, 3, and 4) of DT-CWT are performed, and the correction rate is calculated along with the processing time for each condition. The best 50 pairs of matched points are used to calculate the displacement, and the removal threshold is 10%. Figure 14 shows the ROI images after different levels of DT-CWT. Table 3 shows the error, average processing time of an image, and correction rate at each level.

**Figure 15:**
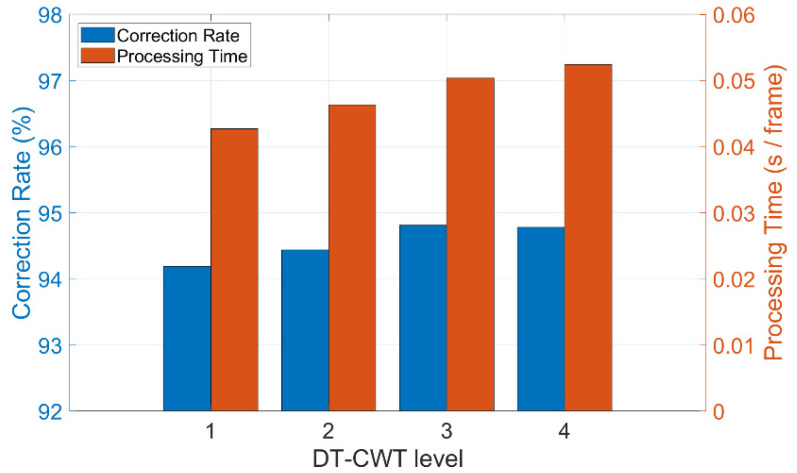
Influences of DT-CWT level on correction rate and processing time.

Figure 15 shows the influences of different DT-CWT levels on the mitigation effect. As the level increases, it takes more time to process a single image, and the correction rate rises as well. Generally, the processing time remains less than 0.06 s per frame, allowing for real-time computation if more powerful CPUs are available and the sampling rate is lower. Therefore, this experiment proves the possibility of real-time monitoring using the proposed method.

Given that DT-CWT at three levels provides the most accurate monitoring and, meanwhile, does not bring a heavy computational burden, the level is set to be three in the following experiments.

After the discussion of the DT-CWT level, the number of best-matched point pairs to be used in calculating the mean displacement is investigated. Different numbers of point pairs are used, and errors are calculated to determine the optimal number of pairs. The numbers of pairs tested and the results are shown in Table 4. Note that 170 is already close to the total number of matched pairs.

**Figure 16:**
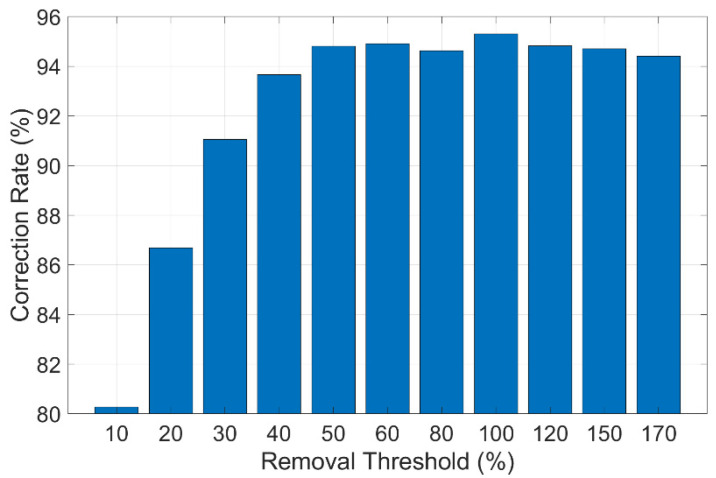
Correction rates at different numbers of point pairs.

**Figure 17:**
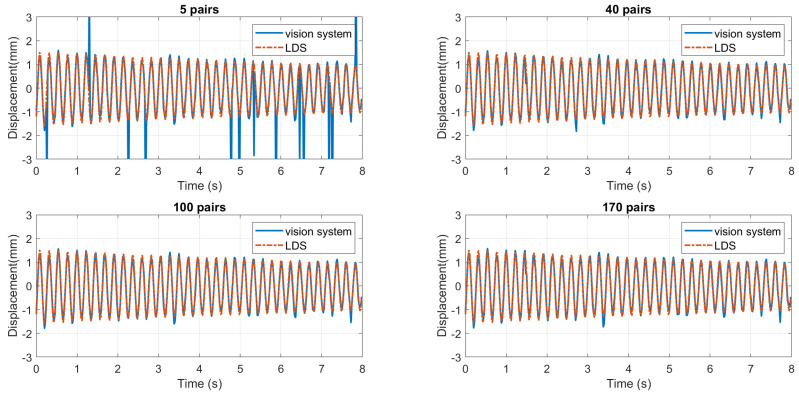
Displacement curves at different numbers of point pairs.

Figure 16 shows the trend of the correction rate as the number of pairs changes. It is clear that when the number of pairs increases, the correction rate first increases and then decreases. The best result occurs when the number is 100. One possible explanation for this tendency is offered here. As stated in Section 2.6, SURF tends to mismatch points that resemble each other in local intensity. Since the feature vector of a mismatched pair generally has a larger SAD, when the number is set to be high, mismatched points may be included in calculating the displacement, thereby adding the error. When the number is low, the averaging effect is undermined, so extreme values can affect the overall displacement significantly. Therefore, the averaging mechanism is most effective when the number of pairs is around the middle. Figure 17 shows the displacement curves at 5, 40, 100, and 170 pairs of points.

**Figure 18:**
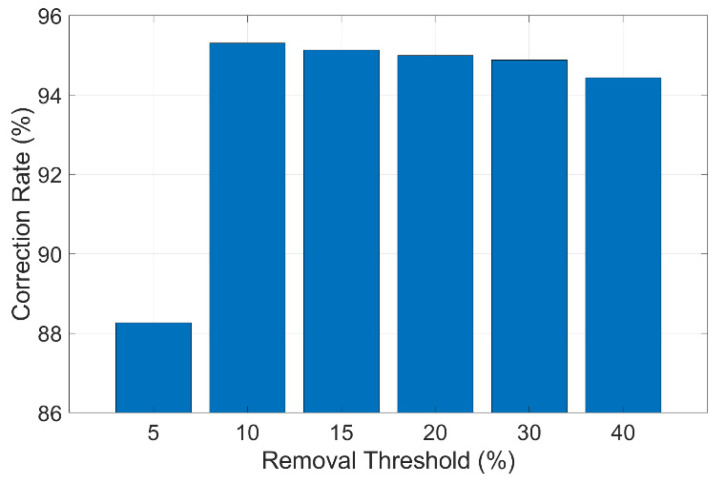
Correction rate at different removal thresholds.

Finally, the impact of the removal threshold is examined. The removal threshold mechanism eliminates a certain portion of point pairs with the highest and lowest displacements, assuming that they are mismatched. If the removal threshold is set to 10%, it means that the pairs with the highest and lowest 10% displacements are excluded from the mean displacement calculation. The best 100 pairs of matched points are used with different removal thresholds, and a similar comparison is carried out. The results are shown in Table 5. Figure 18 shows how the correction rate changes with different removal thresholds. To take a closer look at the results, it can be found that a removal threshold of 10% can produce the most accurate displacements (the correction rate is 95.31%). The reason for this is similar to that explained before. When the threshold is too low, mismatches still exist; when it is too high, the averaging effect is weakened.

It should be pointed out that selecting the best-matched points and introducing the removal threshold both aim to refine the displacement, and their mechanisms are similar. In other experiments conducted by the authors, it was found that the two steps are highly coupled. The proposed method utilizes both of them to ensure the accuracy of monitoring. In practice, the parameters of the two steps need to be tuned according to real conditions.

## 4. Conclusions

This study investigates bridge displacement monitoring based on computer vision in heat haze environments. The properties of heat haze-induced errors are first illustrated, and a heat haze detection method is presented. Meanwhile, a vision-based displacement monitoring technique with heat haze mitigation capability is proposed, and the method of mitigation is elaborated in detail. The proposed method only requires one single image for it to be restored, enabling real-time monitoring. Lastly, static and dynamic experiments are conducted to validate the reliability of the proposed method. Different parameters are discussed in terms of their influences on the mitigation effect. The conclusions are drawn as follows:

1. Heat haze can bring tremendous error to a vision-based displacement monitoring system by distorting the images, which necessitates the mitigation of heat haze-induced errors in long-term monitoring. The distortion caused by heat haze is both spatially and temporally variant. In light of the increased spatial variance of heat haze-distorted images, a heat haze detection method is designed based on SVMs by judging the variance of the intensity of an image. The method achieves an accuracy of 73%.
2. The displacement extraction technique employs SURF to detect the features in the target image. The technique achieves high measurement accuracy (RMSE = 0.0847 mm) in indoor experiments.
3. A heat haze mitigation method using DW-CWT is proposed. For a heat haze-distorted image, the high-frequency components are adjusted through different coefficients iteratively, while the low-frequency components remain intact. After raw displacements are obtained using processed images, a two-step process is proposed to further refine the displacements.
4. The proposed method is validated on a bridge model in the laboratory, where static and dynamic tests are performed. The proposed method achieves a correction rate of 61.05% in the static experiments and 95.31% in the dynamic experiments. It is also demonstrated that the number of points used for calculation and the removal threshold need to be neither too high nor too low to achieve the most accurate results.

However, this study still has some limitations. The heat haze detection method requires a set of images of the target with and without heat haze for training, restricting its transferability. The effectiveness of the proposed method depends highly on the type of markers. Additionally, this study does not include outdoor experiments on real bridges to test the effectiveness of the mitigation method. The authors intend to leave these problems to future research.

## Figures and Tables

**Table 1:** Parameters of the experiment equipment.

Components	Parameters
Camera	Nikon Z30
	Resolution: 1920 × 1080
	Sensor type: CMOS
	Frame rate: 50 fps
Lens	AF-S Nikkor 18–140 mm
	Aperture: F3.5–6.3
Computer	Intel(R) Core i5-1135G7 CPU at 2.40 GHz
	RAM: 16.0 GB
LDS	BOJKE BLG-85N-485
	Sampling rate: 50 Hz
	Precision: 10 μm
	Measurement range: 65–105 mm
Furnace	Gree NSD-12-WG (Gree Electric, Zhuhai, China)
	Maximum power: 1200 W

**Table 2:** Mitigation effects in the static and dynamic experiments.

	Static Experiment		Dynamic Experiment	
	Before	After	Before	After
RMSE (mm)	0.7564	0.2946	0.7248	0.1179
Correction rate	-	61.05%	-	94.81%

**Table 3:** Error, processing time, and correction rate at each level.

Level	1	2	3	4
Processing time (s/frame)	0.0427	0.0463	0.0504	0.0524
RMSE (mm)	0.1219	0.1203	0.1179	0.1181
Correction rate	94.19%	94.44%	94.81%	94.78%

**Table 4:** Errors and correction rates of different numbers of pairs.

**Number of Pairs**	**10**	**20**	**30**	**40**	**50**	**60**
RMSE (mm)	0.2109	0.1699	0.1420	0.1252	0.1179	0.1173
Correction rate	80.28%	86.69%	91.05%	93.67%	94.81%	94.91%
**Number of Pairs**	**80**	**100**	**120**	**150**	**170**	
RMSE (mm)	0.1191	0.1147	0.1178	0.1185	0.1204	
Correction rate	94.63%	95.31%	94.83%	94.72%	94.42%	

**Table 5:** Error and correction rate at different removal thresholds.

Removal Threshold	5%	10%	15%	20%	30%	40%
RMSE (mm)	0.1598	0.1147	0.1159	0.1167	0.1175	0.1203
Correction rate	88.27%	95.31%	95.13%	95.00%	94.88%	94.44%

## Data Availability

The data presented in this study are available on request from the corresponding author due to ongoing research.

## References

[1] Ma Z., Choi J., Sohn H. (2023). Continuous Bridge Displacement Estimation Using Millimeter-Wave Radar, Strain Gauge and Accelerometer. Mech. Syst. Signal Process..

[2] Nassif H.H., Gindy M., Davis J. (2005). Comparison of Laser Doppler Vibrometer with Contact Sensors for Monitoring Bridge Deflection and Vibration. Ndt E Int..

[3] Breuer P., Chmielewski T., Górski P., Konopka E., Tarczyński L. (2015). Monitoring Horizontal Displacements in a Vertical Profile of a Tall Industrial Chimney Using Global Positioning System Technology for Detecting Dynamic Characteristics: GPS, Displacement, Dynamic Characteristics, Measurement Errors. Struct. Control Health Monit..

[4] Han Y., Wu G., Feng D. (2023). Structural Modal Identification Using a Portable Laser-and-Camera Measurement System. Measurement.

[5] Ribeiro D., Calçada R., Ferreira J., Martins T. (2014). Non-Contact Measurement of the Dynamic Displacement of Railway Bridges Using an Advanced Video-Based System. Eng. Struct..

[6] Feng D., Feng M., Ozer E., Fukuda Y. (2015). A Vision-Based Sensor for Noncontact Structural Displacement Measurement. Sensors.

[7] Feng D., Feng M.Q. (2016). Vision-Based Multipoint Displacement Measurement for Structural Health Monitoring: Vision-Based Displacement Measurement for SHM. Struct. Control Health Monit..

[8] Gao X., Ji X., Zhang Y., Zhuang Y., Cai E. (2023). Structural Displacement Estimation by a Hybrid Computer Vision Approach. Mech. Syst. Signal Process..

[9] Xin C., Wang C., Xu Z., Qin M., He M. (2022). Marker-free Vision-based Method for Vibration Measurements of RC Structure under Seismic Vibration. Earthq. Eng. Struct. Dyn..

[10] Nuhman P.A., Singh A., Lambora R., Law M. (2022). Methods to Estimate Subpixel Level Small Motion from Video of Vibrating Cutting Tools. CIRP J. Manuf. Sci. Technol..

[11] Feng D., Feng M.Q. (2018). Computer Vision for SHM of Civil Infrastructure: From Dynamic Response Measurement to Damage Detection—A Review. Eng. Struct..

[12] Zhuang Y., Chen W., Jin T., Chen B., Zhang H., Zhang W. (2022). A Review of Computer Vision-Based Structural Deformation Monitoring in Field Environments. Sensors.

[13] Wang Y.G., Tong W. (2013). A High Resolution DIC Technique for Measuring Small Thermal Expansion of Film Specimens. Opt. Lasers Eng..

[14] Leplay P., Lafforgue O., Hild F. (2015). Analysis of Asymmetrical Creep of a Ceramic at 1350 °C by Digital Image Correlation. J. Am. Ceram. Soc..

[15] Mao Z., Chimitt N., Chan S.H. (2020). Image Reconstruction of Static and Dynamic Scenes Through Anisoplanatic Turbulence. IEEE Trans. Comput. Imaging.

[16] Anantrasirichai N., Achim A., Kingsbury N.G., Bull D.R. (2013). Atmospheric Turbulence Mitigation Using Complex Wavelet-Based Fusion. IEEE Trans. Image Process..

[17] Zhu X., Milanfar P. (2013). Removing Atmospheric Turbulence via Space-Invariant Deconvolution. IEEE Trans. Pattern Anal. Mach. Intell..

[18] Luo L., Feng M.Q. (2017). Vision Based Displacement Sensor with Heat Haze Filtering Capability. Proceedings of the Structural Health Monitoring 2017.

[19] Anantrasirichai N., Achim A., Bull D. (2018). Atmospheric Turbulence Mitigation for Sequences with Moving Objects Using Recursive Image Fusion. Proceedings of the 2018 25th IEEE International Conference on Image Processing (ICIP).

[20] Deledalle C., Gilles J. (2020). Blind Atmospheric Turbulence Deconvolution. IET Image Process..

[21] Luo L., Feng M.Q., Wu J. (2020). A Comprehensive Alleviation Technique for Optical-turbulence-induced Errors in Vision-based Displacement Measurement. Struct. Control Health Monit..

[22] Selesnick I.W., Baraniuk R.G., Kingsbury N.C. (2005). The Dual-Tree Complex Wavelet Transform. IEEE Signal Process. Mag..

[23] Bay H., Ess A., Tuytelaars T., Van Gool L. (2008). Speeded-Up Robust Features (SURF). Comput. Vis. Image Underst..

[24] Dworakowski Z., Kohut P., Gallina A., Holak K., Uhl T. (2016). Vision-Based Algorithms for Damage Detection and Localization in Structural Health Monitoring: Vision-Based Algorithms for Damage Detection and Localization. Struct. Control Health Monit..

[25] Michael C.R., Byron M.W. (2018). Imaging Through Turbulence.

[26] Gal R., Kiryati N., Sochen N. (2014). Progress in the Restoration of Image Sequences Degraded by Atmospheric Turbulence. Pattern Recognit. Lett..

[27] He K., Sun J., Tang X. (2011). Single Image Haze Removal Using Dark Channel Prior. IEEE Trans. Pattern Anal. Mach. Intell..

[28] Luo L., Feng M.Q., Wu J., Bi L. (2021). Modeling and Detection of Heat Haze in Computer Vision Based Displacement Measurement. Measurement.

[29] Lowe D.G. (2004). Distinctive Image Features from Scale-Invariant Keypoints. Int. J. Comput. Vis..

[30] Juan L., Gwun O. (2009). A Comparison of SIFT, PCA-SIFT and SURF. Int. J. Image Process..

[31] Viola P., Jones M. Rapid Object Detection Using a Boosted Cascade of Simple Features. Proceedings of the Proceedings of the 2001 IEEE Computer Society Conference on Computer Vision and Pattern Recognition. CVPR 2001.

[32] Heinly J., Dunn E., Frahm J.-M. (2012). Comparative Evaluation of Binary Features. Proceedings of the Computer Vision—ECCV 2012.

[33] Durga R.V., Kumari O., Prakash M.S., Kumar P.D., Tirupathi Y. (2014). Region-Based Image Fusion Using Complex Wavelets. IOSR J. Electron. Commun. Eng..

[34] Lewis J.J., O’Callaghan R.J., Nikolov S.G., Bull D.R., Canagarajah N. (2007). Pixel- and Region-Based Image Fusion with Complex Wavelets. Inf. Fusion.

[35] Wan T., Canagarajah N., Achim A. (2009). Segmentation-Driven Image Fusion Based on Alpha-Stable Modeling of Wavelet Coefficients. IEEE Trans. Multimed..

